# Metabolomic Profiling of Fungal Pathogens Responsible for Root Rot in American Ginseng

**DOI:** 10.3390/metabo10010035

**Published:** 2020-01-14

**Authors:** Natasha DesRochers, Jacob P. Walsh, Justin B. Renaud, Keith A. Seifert, Ken K.-C. Yeung, Mark W. Sumarah

**Affiliations:** 1 London Research and Development Centre, Agriculture and Agri-Food Canada, 1391 Sandford St., London, ON N5V 4T3, Canada; natasha.desrochers5@canada.ca (N.D.); jwalsh69@uwo.ca (J.P.W.); justin.renaud@canada.ca (J.B.R.); 2 Department of Chemistry, University of Western Ontario, 1151 Richmond St., London, ON N6A 3K7, Canada; kyeung@uwo.ca; 3 Ottawa Research and Development Centre, Agriculture and Agri-Food Canada, 960 Carling Ave., Ottawa, ON K1A 0C6, Canada; Keith.seifert@carleton.ca; 4 Department of Biochemistry, University of Western Ontario, 1151 Richmond St., London, ON N6A 3K7, Canada

**Keywords:** American ginseng, *Ilyonectria*, root rot, metabolomics, molecular networking, GNPS

## Abstract

Ginseng root is an economically valuable crop in Canada at high risk of yield loss caused by the pathogenic fungus *Ilyonectria mors-panacis,* formerly known as *Cylindrocarpon destructans*. While this pathogen has been well-characterized from morphological and genetic perspectives, little is known about the secondary metabolites it produces and their role in pathogenicity. We used an untargeted tandem liquid chromatography-mass spectrometry (LC-MS)-based approach paired with global natural products social molecular networking (GNPS) to compare the metabolite profiles of virulent and avirulent *Ilyonectria* strains. The ethyl acetate extracts of 22 *I. mors-panacis* strains and closely related species were analyzed by LC-MS/MS. Principal component analysis of LC-MS features resulted in two distinct groups, which corresponded to virulent and avirulent *Ilyonectria* strains. Virulent strains produced more types of compounds than the avirulent strains. The previously reported *I. mors-panacis* antifungal compound radicicol was present. Additionally, a number of related resorcyclic acid lactones (RALs) were putatively identified, namely pochonins and several additional derivatives of radicicol. Pochonins have not been previously reported in *Ilyonectria* spp. and have documented antimicrobial activity. This research contributes to our understanding of *I. mors-panacis* natural products and its pathogenic relationship with ginseng.

## 1. Introduction

Ginseng root has long been used as a traditional oriental medicine. This is evidenced by the global ginseng market, valued at over two billion dollars [1]. The main species cultivated in Canada is *Panax quinquefolius*, or American ginseng [2]. Although it is valuable, selling at approximately $55/kilogram, it is a high-risk crop because of the demands of its growth conditions [2,3]. Seeds germinate for two years and plants typically grow for four years before harvest occurs [2]. Ginseng cultivation requires cool temperatures, low light, and moist soil [2]. Unfortunately, these are ideal growth conditions for several phylogenetically-delimited species of fungal pathogens, all formerly classified in a broadly-defined species called *Cylindrocarpon destructans* but now classified in the genera *Ilyonectria* and *Neonectria* [4,5]. *Ilyonectria* pathogens cause root rot and rusty root disease in ginseng and contribute to average crop losses of 20–30% at time of harvest [2,6]. The segregate species within *Ilyonectria* have varying degrees of virulence toward ginseng root. Aggressive species are responsible for disappearing root rot while less aggressive strains cause rusty root disease [4,7]. Root rot is the primary concern for ginseng farmers; it can infect ginseng plants at any stage of growth, making it difficult to control [8,9]. Because of its persistence and virulence, root rot is a contributor to ginseng replant disease, another major concern for ginseng farmers [10]. Replant disease occurs when ginseng planted in the same soil as a previous ginseng crop fails to grow. This is a multifactorial issue, influenced by accumulation of phytotoxic compounds in the soil from the ginseng plants themselves, and by changes to the soil microbiome over time.

While many studies have examined morphological and genetic characteristics of fungal strains causing the disease, few have focused on the secondary metabolites made by these species. A handful of metabolites have been previously identified in extracts from *Ilyonectria* and *Neonectria* strains isolated from ginseng. These include the antifungal compound radicicol and the antiviral brefeldin A [11]. Radicicol is potently antifungal against several species of pathogenic fungi that inhabit the same environment as ginseng root pathogens [11]. Additionally, we recently isolated the iron-chelating molecule *N*,*N*′,*N*″-Triacetylfusarinine C (TAFC) from liquid media extracts of *I. mors-panacis* [12]. TAFC allows fungi to scavenge iron from the extracellular environment [13]. Apart from these, very few other metabolites have been reported from fungal species causing root diseases in ginseng, and we do not have a clear picture of what role secondary metabolites play in the development of root rot disease. 

Screening by liquid chromatography high resolution mass spectrometry (LC-HRMS) provides a powerful tool to identify secondary metabolites from agriculturally relevant fungal species, which can lead to a deeper understanding of their role in disease. Additionally, tandem HRMS data can be used to create molecular networks, which assist in identifying groups of structurally related compounds [14]. The online platform global natural products social (GNPS) molecular networking is one of the most frequently used means of analyzing mass spectra-based molecular networks because it allows for the comparison of mass spectra to large libraries of standards and to user-uploaded datasets [15]. 

Here, we present a non-targeted metabolomics approach to screening virulent and avirulent strains of *Ilyonectria* and *Neonectria*. We used LC-HRMS data analyzed by Principal Component Analysis (PCA) to confirm that virulent species have distinct metabolic profiles from those of avirulent strains. This was coupled with GNPS molecular networking of tandem HRMS data to identify classes of secondary metabolites distinct to fungal strains that cause ginseng root rot. 

## 2. Results

### 2.1. Principal Component Analysis of LC-HRMS Data

In this study, we chose *Ilyonectria* and *Neonectria* strains that were previously investigated by both Seifert et al. [6] and Cabral et al. [5]. These strains have taxonomic data and information about their virulence towards ginseng roots, whether by a direct pathogenicity assay, or based on their phylogenetic relatedness to assayed strains [6,12]. Strains that are virulent and avirulent towards ginseng roots were analyzed by tandem HRMS to determine if they have distinct metabolic profiles. 

**Figure 1:**
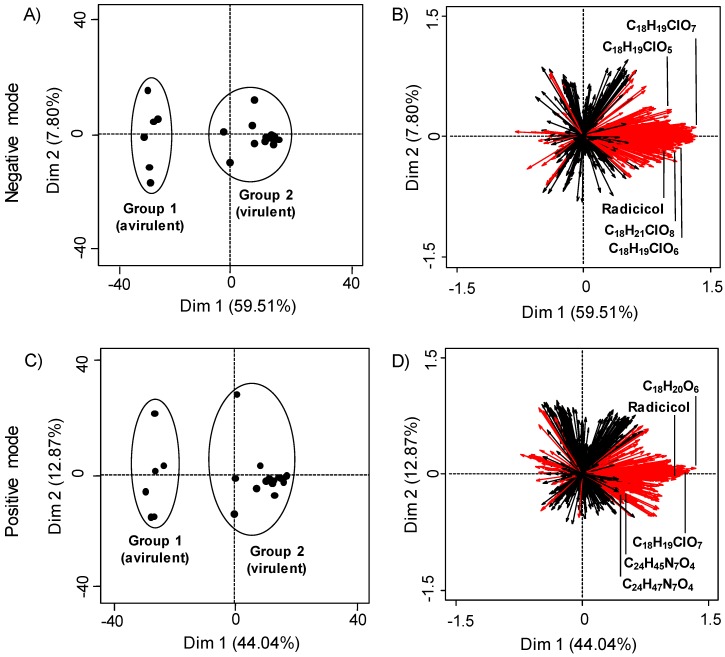
Principal component analysis (PCA) plots of metabolites from *Ilyonectria* and *Neonectria* spp. in both positive and negative ionization modes. Plots (**A**,**C**) show individual samples with group 1 being avirulent strains and group 2 containing virulent strains. Plots (**B**,**D**) show the factor loadings for samples, with each ray representing an LC-MS feature. Red rays indicate features that significantly contribute to PCA distribution (*p* < 0.05 Kruskal-Wallis test with Benjamini-Hochberg *p* value correction). The five most abundant compounds produced by virulent strains are labeled.

PCA was performed using metabolite peak areas acquired with LC-HRMS in both negative and positive ionization modes. In positive ionization mode, 1813 features were detected and in negative ionization mode, 1019 features were detected using *xcms* (parameters described in Supplementary Text S1). PCA plots (Figure 1) were generated, to visually represent the degree of similarity or difference between strains based on the identities and relative quantities of metabolites. In both ionization modes, two distinct groups were observed. Group 1 comprised strains DAOMC 144524, 226721, 226730, 251608, 251609, and strain 94-1356, all of which are avirulent. Group 2 comprised strains DAOMC 139398, 221059, 226727, 226729, 230337, 230338, 234582, 251601, 251602, 251603, 251604, 251605, 251606, 251607, 251610, and 251611, all of which are virulent (Figure 1). 

### 2.2. Determining LC-MS Features Significant to Virulent Strains

To determine which metabolites influence the division of virulent and avirulent strains, a Kruskal-Wallis test for non-parametric data was performed. Consequently, 416 and 418 features were significantly different in negative and positive ionization mode, respectively (*p* < 0.05). These are highlighted red in factor loadings plots (Figure 1B,D). In both ionization modes, the majority of significant metabolites were produced in higher quantities by virulent strains, predominantly *I. mors-panacis*, as well as two *I. robusta* strains.

In both ionization modes, the most abundant metabolite by peak area was radicicol (C_18_H_17_ClO_6_), confirmed by comparing to experimental ESI-Q-TOF MS/MS data retrieved from METLIN [16,17]. Radicicol was only produced by virulent strains. The five most abundant features detected in extracts of virulent strains were subsequently examined in both ionization modes. Table 1 below displays their accurate *m*/*z* and chemical formulas, additional abundant metabolites in both positive and negative mode are presented Supplementary Tables S1 and S2. Because of the similarities among several of the chemical formulas and to further explore the data, tandem HRMS spectra were analyzed by molecular networking with GNPS to ascertain whether these represented classes of compounds. Neither brefeldin A nor TAFC were present in either ionization mode under the conditions tested.

### 2.3. Molecular Networking with GNPS

**Figure 2:**
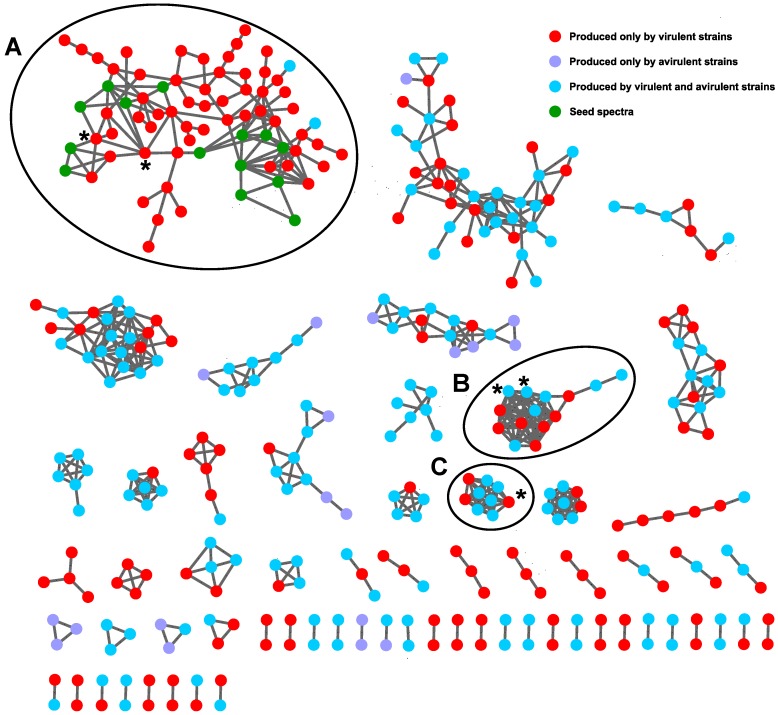
GNPS molecular network of metabolites from strains of *Ilyonectria* and *Neonectria* grown on potato dextrose agar. Nodes represent features detected by tandem HRMS (positive ionization mode), and lines represent cosine similarity scores above 0.6. The five most abundant compounds produced by virulent strains are found within clusters A, B, and C, and are indicated by (*).

Tandem HRMS data collected in positive ionization mode were analyzed with the GNPS molecular networking portal and visualized in Cytoscape [18]. A network was also created with negative mode data, but it was of poorer quality than the positive mode network (indicated by a low number of total nodes and of connected nodes) and was not explored further. The MSCluster algorithm converts each MS/MS spectrum into a vector, then compares vectors using a dot-product calculation [14]. The similarity between spectra is given by the cosine between the two vectors, with 1 being identical and 0 being unrelated. Cosine cut-off scores are typically set between 0.5 to 0.7 [19]. The cosine similarity score was set to 0.6, therefore nodes that are connected have spectral similarity greater than 0.6. Nodes represent individual features and lines connecting them indicate similarity related to shared fragments. To simplify the network, features from media and clusters comprised solely of seed spectra were removed. Seed spectra included radicicol and two other unidentified compounds purified from extracts of DAOMC 251601 with chemical formulas of C_18_H_20_O_5_ and C_18_H_20_O_6_ (Figure 2). 

**Figure 3:**
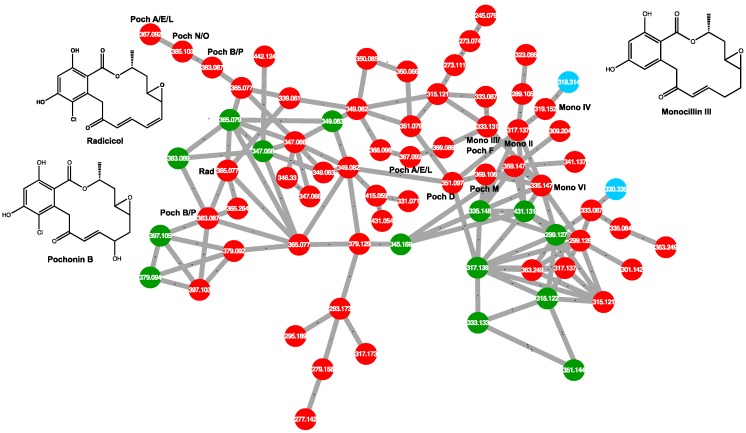
Close-up of the GNPS molecular network cluster containing radicicol and related compounds. *m*/*z* values are labeled on nodes, and putative compound identifications are assigned where applicable. Structures are included for compounds from Table 1 that are found within this cluster. (Poch—pochonin; Mono—monocillin; Rad—radicicol)**.**

In Figure 2, the five most abundant compounds from Table 1 are divided across three clusters and are highlighted by asterisks (*). Cluster A contains radicicol and a compound with the formula C_18_H_19_ClO_7_. Cluster B contains compounds with formulas of C_28_H_49_NO_6_ and C_28_H_51_NO_6_. Neither compound was identifiable under these experimental conditions. While the Kruskal-Wallis test determined which features were statistically different between virulent and avirulent strains, it did not identify compounds unique to either group. The test simply identified which group produced them in higher abundance. By colouring the GNPS output according to the features unique to each group, it became evident that the compounds found within cluster B were produced by both virulent and avirulent strains and therefore they were not pursued for further identification. Cluster C only contains one of the five most abundant compounds: C_18_H_20_O_6_. The node is a sodiated peak, and although it sits apart from the other compounds from Table 1, its [M + H]^+^ peak is found within cluster A. Because cluster A is comprised almost exclusively of features that were only detected in extracts of virulent strains (red nodes), it was investigated more closely to establish a metabolomic profile of virulent strains. The cluster includes several chlorine-containing compounds with chemical formulas similar to that of radicicol. They are connected to one another, indicating they share similar fragmentation spectra. Cluster A is pictured on its own in Figure 3.

**Figure 4:**
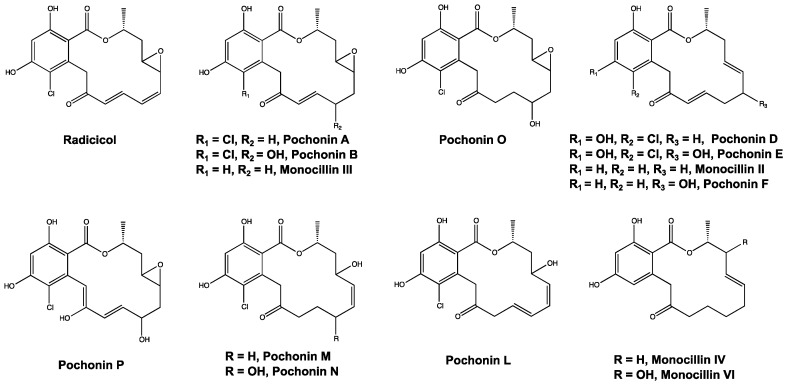
Structures for radicicol and other putatively identified resorcyclic acid lactones from extracts of *Ilyonectria mors-panacis* and *Ilyonectria robusta*.

Several compounds are made solely by virulent strains of *Ilyonectria* and have chemical formulas matching those of reported resorcyclic acid lactones (RALs) including pochonins and monocillins, which are structurally related to radicicol. For example, from Table 1, the compounds with chemical formulas of C_18_H_20_O_6_ and C_18_H_19_ClO_7_ match the formulas for monocillin III and pochonin B, respectively. Within the network, radicicol from extracts of aggressive strains is connected with a compound tentatively identified as Pochonin B. They share a cosine similarity score of 0.82, indicating a high degree of spectral similarity, which supports this identification. All putatively identified pochonins and monocillins are labeled in Figure 3 and are shown in Figure 4. Some nodes in Figure 3 are labeled with multiple putative identifications, because some pochonins and monocillins share chemical formulas and *m*/*z* values. 

## 3. Discussion

Fungal strains were chosen for analysis that had been previously assayed on ginseng to assess their virulence. These comprised a mix of *Ilyonectria* species and a single strain of *Neonectria*, included as an outgroup control. Using PCA, we established that virulent strains of *Ilyonectria* capable of causing ginseng root rot produced a chemical profile distinct from strains that cannot cause root rot. Notably, there was no difference in the type or abundance of metabolites made by strains of *I. mors-*panacis isolated from either American and Asian ginseng. This is consistent with previous reports that *I. mors-panacis* isolates have high genetic similarity, regardless of geographic origin [5,6]. There was also no difference in the chemical profiles of *I. mors-panacis* or *I. robusta*, perhaps indicating that secondary metabolites are important to the virulence of these species toward ginseng root.

Overall, avirulent strains produced a smaller variety of compounds, and in lower relative quantities compared to virulent strains. Although avirulent strains include four species, there was little difference in the types of compounds made by this group. The list of the most abundant compounds was slightly different for positive versus negative ionization mode. The discrepancy is probably a consequence of some compounds ionizing preferentially in one mode over the other. Despite the difference, both sets of factor loadings plots demonstrate that overall, aggressive strains release higher quantities of compounds into extracellular media than less aggressive strains. With respect to the absence of brefeldin A and TAFC, it is hypothesized that the growth conditions in this experiment did not favour their production, as the cultures in this study were grown on solid media, whereas TAFC and brefeldin A have predominantly been observed in liquid culture [12,20]. We were unable to identify any of the statistically significant compounds made by avirulent strains.

While PCA is useful in metabolomics for determining overall similarity or difference, it is challenging to rely solely on PCA to identify significant compounds. Feature lists are often too large to examine manually and it is difficult to identify compounds given only their *m*/*z* value or chemical formula. GNPS molecular networking supplements PCA by providing information about the structural similarity among compounds in a sample, allowing for identification of compound classes. In this case, it allowed the identification of several pochonins and monocillins (Figure 4), which are structurally similar to radicicol. Pochonins and monocillins have been well-documented in other species of the same taxonomic order as *I. mors-panacis*, the Hypocreales, such as *Pochonia chlamydosporia* and *Monocillium nordinii* (teleomorph *Niesslia*) and have antimicrobial effects against several organisms [21,22,23]. Antimicrobial RALs may indirectly assist *I. mors-panacis* and *I. robusta* in colonizing ginseng roots, by acting against other pathogens inhabiting the same soil environment [11]. This may give root rot pathogens an improved chance of surviving in the soil, and of eventually infecting ginseng.

PCA and GNPS molecular networking allow for rapid analysis of complex mixtures and putative identification of compounds, as well as identification of significant molecular classes produced by organisms. However, these tools are somewhat limited in their ability to confidently identify compounds without the use of standards. As seen in Figure 3, several compounds may share the same *m*/*z* and are therefore not discernable from one another without comparison to standards. This may be done by analyzing standards alongside a set of samples, or by comparison to a published spectrum. Unidentified compounds must be isolated in sufficient quantities for characterization by nuclear magnetic resonance spectroscopy (NMR). Molecular networking, though limited in this regard, is still a powerful tool for the initial identification of compounds and for identifying target compounds for further isolation and characterization. In this study, it identified a class of compounds only produced by aggressive strains of *Ilyonectria*. Analysis by PCA and molecular networking differentiated virulent and avirulent strains of *Ilyonectria* and *Neonectria* based on their metabolites. This work guides us toward virulent strains of *Ilyonectria* for the isolation and characterization of their secondary metabolites. The RALs produced by *Ilyonectria* should be tested in future experiments as pathogenicity factors, to determine if they play a role in the virulence of ginseng root rot. 

## 4. Materials and Methods 

### 4.1. Cell Culture and Secondary Metabolite Extraction

All isolates are deposited in the Canadian Collection of Fungal Cultures (DAOMC), except for the strain of *Neonectria obtusisporum* 94-1356, from the laboratory collection of K.A.S. The 22 isolates of *Ilyonectria* and *Neonectria* spp. (Table 2) were inoculated on Potato Dextrose Agar (PDA) (2% (*w*/*v*) agar (Fisher Scientific, Fair Lawn, NJ, USA), 2.4% (*w*/*v*) Potato Dextrose Broth powder (Sigma-Aldrich, St. Louis, MO, USA), and distilled water) and was supplemented with 3.47 × 10^−5^ M zinc sulfate heptahydrate (Sigma-Aldrich) and 3.13 × 10^−5^ M copper sulfate (Sigma-Aldrich). 

After 20 days of growth in darkness at 21 °C the plates were extracted as per the method outlined by Smedsgaard [24], with the following changes: the number of plugs was increased to six, plugs were extracted with 4 mL of ethyl acetate, extracts were dried by centrifugal vacuum evaporation, dried extracts were reconstituted in 1 mL of 3:1 methanol:water, and were filtered through a 0.45 µm PTFE filter (Chromatographic Specialties, Inc., Brockville, ON K6V 5W1, CA). Composite quality control (QC) samples were made by combining 50 µL from each completed extract.

### 4.2. LC-MS Experiments

High resolution MS (HRMS) and high-resolution MS/MS (HRMS/MS) analysis of secondary metabolites was performed using a Q-Exactive Orbitrap mass spectrometer (Thermo Fisher Scientific, Waltham, MA, USA) with a heated electrospray ionization (HESI) source connected in tandem to an Agilent 1290 ultra-high-performance liquid chromatography (UHPLC) system (see Data S1 for raw data). Positive and negative ionization modes were assessed for both HRMS and HRMS/MS. Separation was performed with a dual-solvent system with 0.1% (*v*/*v*) formic acid in water and 0.1% (*v*/*v*) formic acid in acetonitrile (solvents A and B, respectively), at a flow rate of 0.3 mL/min. The gradient was held at 0% B for 0.5 min, increased to 100% B over 3 min, held at 100% B for 2.5 min, decreased to 0% B over 0.5 min and finally held at 0% B for one minute. For each sample, 5 µL was injected onto an EclipsePlus RRHD C-18 column (2.1 × 50 mm, 1.8 µm; Agilent) heated to 35 °C. HESI conditions were as follows: capillary temperature, 400 °C; sheath gas, 19 units (positive ionization mode) and 17 units (negative ionization mode); auxiliary gas, 8 units; probe heater temperature, 450 °C; S-Lens RF level, 45; capillary voltage, 3.9 kV (positive ionization mode) and 4.0 kV (negative ionization mode). 

All samples were analyzed by HRMS with the following settings: resolution, 140,000; automatic gain control (AGC) target, 5 × 10^5^; maximum injection time, 512 ms; scan range, *m*/*z* 80–1200. Quality control (QC) samples were injected at the beginning, end, and periodically throughout the run to assess instrument drift and batch effect [25]. Samples were also analyzed by tandem HRMS, with the following settings: resolution, 17,500; AGC target, 1 × 10^6^; max IT, 64 ms; scan range, 200–2000 Da; normalized collision energy (NCE), 35. 

### 4.3. LC-MS Data Processing

For principle component analysis (PCA), Thermo Raw HRMS files were converted into centroid mode using MSConvert [26] and filtered with peak picking set at MS level 1 (Vendor algorithm). This returned mzML files that are usable in R (r-project.org). The mzML files were processed in R 3.5.3 with the software package *xcms* 3.4.4 [27,28,29] (parameters listed in Supplementary Text S1). All zero values were imputed with two-thirds of the lowest value measured per metabolite [30], metabolites that were only found in blank media were excluded, and isotopic peaks were removed manually. Peak area values were log10-transformed and pareto scaled [31], then PCA was performed with the R packages *FactoMineR* 1.41 [32] and *MetabolAnalyze* 1.3 [33,34]. Additionally, factor loadings values were calculated with the same packages listed above. The first and second principal component scores were plotted against each other. Positive and negative ionization modes were analyzed separately. Quality control samples were included in preliminary PCA plots (Supplementary Figure S1), and it was determined that there was little influence from batch effect or instrument drift. A Kruskal-Wallis test for non-parametric data was performed on metabolite peak areas from the virulent and avirulent groups using R, and Bejamini-Hochberg *p*-value correction was applied. Metabolites with a corrected *p*-value < 0.05 were considered to be significantly different between groups.

### 4.4. Molecular Networking Parameters and Visualization

For molecular networking in GNPS, Thermo Raw HRMS files analyzed in positive ionization mode were converted to mzML format with MSConvert, peak picking from MS level 1–2, 32-bit binary encoding precision, and no compression. Files were uploaded to GNPS and analyzed with the following parameters: precursor ion mass tolerance, 0.001 Da; fragment ion mass tolerance, 0.001 Da; min pairs cosine score, 0.6; Network TopK, 10; maximum connected component size, 100; minimum matched fragment ions, 4; minimum cluster size, 2, and MSCluster, on. Three in-house MS/MS files of radicicol and putatively related compounds purified from extracts of *I. mors-panacis* strain DAOMC 251601 were included as seed spectra. These have chemical formulas of C_18_H_20_O_5_ and C_18_H_20_O_6_. The output was downloaded as a GRAPHML file and visualized in Cytoscape 2.6.1 [18]. For simplification, all features that were only present in the blank media and clusters comprised of only seed spectra were removed from the network.

## Figures and Tables

**Table 1:** The five most abundant compounds produced by virulent and avirulent strains of *Ilyonectria* grown on potato dextrose agar in positive and negative ionization modes. (*) indicates radicicol, the most abundant compound in both ionization modes.

Positive		Negative	
*m*/*z*	Formula	*m*/*z*	Formula
355.115 [M + Na]^+^	C_18_H_20_O_6_	349.0847 [M − H]^−^	C_18_H_19_ClO_5_
* 365.0785 [M + H]^+^	C_18_H_17_ClO_6_	* 363.0641 [M − H]^−^	C_18_H_17_ClO_6_
383.0890 [M + H]^+^	C_18_H_19_ClO_7_	365.0795 [M − H]^−^	C_18_H_19_ClO_6_
496.3631 [M + H]^+^	C_28_H_49_NO_6_	381.0746 [M − H]^−^	C_18_H_19_ClO_7_
498.3787 [M + H]^+^	C_28_H_51_NO_6_	399.085 [M − H]^−^	C_18_H_21_ClO_8_

**Table 2:** Isolates of *Ilyonectria* and *Neonectria* spp. used in this study. All cultures except (*) were received from the Canadian Collection of Fungal Cultures (DAOMC). (*) is not deposited in the DAOMC at the time of writing and is from the laboratory collection of K.A.S.

ID	Species	Host	Origin
139398	*Ilyonectria robusta*	*Prunus cerasus* (Sour cherry)	Canada, ON
144524	*I. torresensis*	*Vitis vinifera* (Grape)	ON
220159	*I. mors-panacis*	*Panax quinquefolius* (American ginseng)	ON
226721	*I. rufa*	*Pseudotsuga menziesii* (Douglas fir)	Canada, BC
226727	*I. mors-panacis*	*P. quinquefolius*	ON
226729	*I. robusta*	*P. quinquefolius*	ON
226730	*I. estremocensis*	*Picea glauca* (White spruce)	Canada, QC
230337	*I. mors-panacis*	*Panax ginseng* (Asian ginseng)	Japan
230338	*I. mors-panacis*	*P. ginseng*	Japan
234582	*I. mors-panacis*	*P. quinquefolius*	ON
251601	*I. mors-panacis*	*P. quinquefolius*	ON
251602	*I. mors-panacis*	*P. quinquefolius*	ON
251603	*I. mors-panacis*	*P. quinquefolius*	ON
251604	*I. mors-panacis*	*P. quinquefolius*	ON
251605	*I. mors-panacis*	*P. quinquefolius*	ON
251606	*I. mors-panacis*	*P. quinquefolius*	ON
251607	*I. mors-panacis*	*P. quinquefolius*	ON
251608	*I. rufa*	*P. menziesii*	BC
251609	*I. rufa*	*P. glauca*	QC
251610	*I. mors-panacis*	*P. quinquefolius*	ON
251611	*I. mors-panacis*	*P. quinquefolius*	ON
94-1356 *	*Neonectria obtusisporum*	*Picea mariana* (Black spruce)	QC

## Supplementary Materials

The following are available online at https://www.mdpi.com/2218-1989/10/1/35/s1, Figure S1: PCA plots with QC samples included. (A) negative ionization mode. (B) positive ionization mode, Table S1: Most abundant metabolites by peak area in positive ionization mode, produced by virulent strains of *Ilyonectria*, Table S2: Most abundant metabolites by peak area in negative ionization mode, produced by virulent strains of *Ilyonectria.* Text S1: Parameters for xcms processing, Data S1: These data are available at the NIH Common Fund’s National Metabolomics Data Repository (NMDR) website, the Metabolomics Workbench, https://www.metabolomicsworkbench.org where they have been assigned Project ID PR000875. The data can be accessed directly via the Project DOI: 10.21228/M8KM4W. This work is supported by NIH grant U2C-DK119886 [35].
