# Supplementary material for: Metabolomic Profiling of Fungal Pathogens Responsible for Root Rot in American Ginseng
Source: Metabolites. 2020 Jan 14;10(1):35. doi: 10.3390/metabo10010035 (PMC7023087; doi:10.3390/metabo10010035)
Supplement: Supplementary file 1 [file metabolites-10-00035-s001.pdf]

Supplementary Materials:

Text S1. Parameters for *xcms* processing

xSet function: method="centWave"; prefilter=c(5,5000), ppm=5, snthresh=10, peakwidth=c(5,30), noise=1000000.

Group function: bw=5, minfrac=0.001, mzwid=0.015.

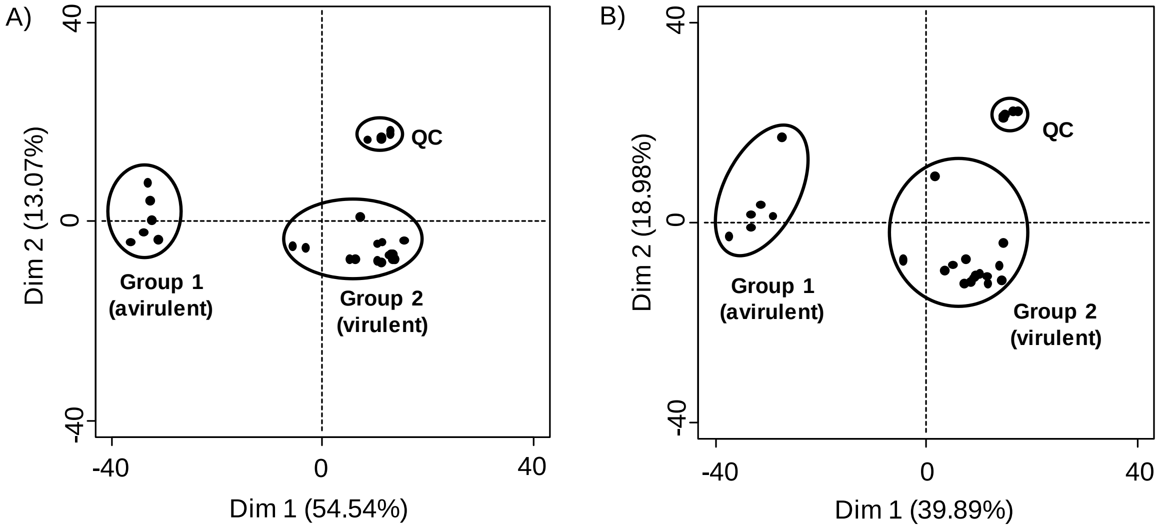

Figure S1. PCA plots with QC samples included. A) negative ionization mode. B) positive ionization mode.

Table S1. Twenty most abundant metabolites by peak area in positive ionization mode, produced by virulent strains of *Ilyonectria*. (Significantly different from avirulent strains by a Kruskal-Wallis test with Benjamini-Hochberg p value correction, $p < 0.05$).

m/z $[M+H]^+$	Rt (min)	Formula	p-value
205.1951	4.43	$C_{15}H_{24}$	0.002
205.1951	3.55	$C_{15}H_{24}$	0.011
221.1899	4.47	$C_{15}H_{24}O$	0.003
233.0807	2.77	$C_{13}H_{12}O_4$	0.002
235.0964	2.68	$C_{13}H_{14}O_4$	0.005
253.1796	4.38	$C_{15}H_{24}O_3$	0.002
333.133	3.17	$C_{18}H_{20}O_6$	0.002
349.0834	2.99	$C_{18}H_{17}ClO_5$	0.002
351.0992	3.67	$C_{18}H_{19}ClO_5$	0.002
365.0785	3.19	$C_{18}H_{17}ClO_6$	0.002
365.0786	2.65	$C_{18}H_{17}ClO_6$	0.002
367.0937	3.32	$C_{18}H_{19}ClO_6$	0.002
383.089	2.92	$C_{18}H_{19}ClO_7$	0.002
383.0891	2.57	$C_{18}H_{19}ClO_7$	0.002
388.2115	3.02	$C_{22}H_{29}NO_5$	0.011
406.105	3.19	$C_{20}H_{21}NClO_6$	0.002
496.3631	3.74	$C_{24}H_{45}N_7O_4$	0.031
498.3787	3.93	$C_{24}H_{47}N_7O_4$	0.049
500.3943	4.17	$C_{24}H_{49}N_7O_4$	0.031
518.3241	3.62	$C_{29}H_{39}N_7O_2$	0.002

Table S2. Twenty most abundant metabolites by peak area in negative ionization mode, produced by virulent strains of *Ilyonectria*. (Significantly different from avirulent strains by a Kruskal-Wallis test with Benjamini-Hochberg p value correction, $p < 0.05$).

m/z $[M-H]^-$	Rt (min)	Formula	p-value
201.1124	2.45	$C_{10}H_{18}O_4$	0.001
259.0610	3.07	$C_{14}H_{12}O_5$	0.002
275.0561	2.94	$C_{29}H_{51}NO_8$	0.002
275.1864	2.72	$C_{14}H_{28}O_5$	0.024
349.0847	3.67	$C_{18}H_{19}ClO_5$	0.001
363.0641	3.19	$C_{18}H_{17}ClO_6$	0.001
363.0641	3.65	$C_{18}H_{17}ClO_6$	0.001
365.0795	2.99	$C_{18}H_{19}ClO_6$	0.001
367.0951	3.34	$C_{18}H_{21}ClO_6$	0.001
379.0588	3.18	$C_{18}H_{17}ClO_7$	0.001
379.0590	2.95	$C_{18}H_{17}ClO_7$	0.001
381.0746	2.74	$C_{18}H_{19}ClO_7$	0.001
383.0899	2.87	$C_{18}H_{21}ClO_7$	0.001
383.0903	3.21	$C_{18}H_{21}ClO_7$	0.001
393.1111	4.08	$C_{20}H_{23}ClO_6$	0.001
397.0694	2.74	$C_{18}H_{19}ClO_8$	0.001
399.0851	2.65	$C_{18}H_{21}ClO_8$	0.001
526.0942	2.88	$C_{23}H_{24}NSClO_9$	0.001
540.3541	3.74	$C_{29}H_{51}NO_8$	0.024
542.3698	3.93	$C_{29}H_{53}NO_8$	0.030
